# Innate immune remodeling by short‐term intensive fasting

**DOI:** 10.1111/acel.13507

**Published:** 2021-10-27

**Authors:** Jiawei Qian, Yixuan Fang, Na Yuan, Xueqin Gao, Yaqi Lv, Chen Zhao, Suping Zhang, Quan Li, Lei Li, Li Xu, Wen Wei, Jianrong Wang

**Affiliations:** ^1^ Research Center for Blood Engineering and Manufacturing Cyrus Tang Medical Institute National Clinical Research Center for Hematologic Diseases Collaborative Innovation Center of Hematology Jiangsu Institute of Hematology Institute of Blood and Marrow Transplantation The First Affiliated Hospital of Soochow University State Key Laboratory of Radiation Medicine and Protection Soochow University Suzhou China; ^2^ Soyo Center Soochow University Suzhou China; ^3^ Suzhou Ninth Hospital affiliated to Soochow University Suzhou China

**Keywords:** fasting, human, innate immunity, longevity, neutrophils

## Abstract

Previous studies have shown that long‐term light or moderate fasting such as intermittent fasting can improve health and prolong lifespan. However, in humans short‐term intensive fasting, a complete water‐only fasting has little been studied. Here, we used multi‐omics tools to evaluate the impact of short‐term intensive fasting on immune function by comparison of the CD45^+^ leukocytes from the fasting subjects before and after 72‐h fasting. Transcriptomic and proteomic profiling of CD45^+^ leukocytes revealed extensive expression changes, marked by higher gene upregulation than downregulation after fasting. Functional enrichment of differentially expressed genes and proteins exposed several pathways critical to metabolic and immune cell functions. Specifically, short‐term intensive fasting enhanced autophagy levels through upregulation of key members involved in the upstream signals and within the autophagy machinery, whereas apoptosis was reduced by down‐turning of apoptotic gene expression, thereby increasing the leukocyte viability. When focusing on specific leukocyte populations, peripheral neutrophils are noticeably increased by short‐term intensive fasting. Finally, proteomic analysis of leukocytes showed that short‐term intensive fasting not only increased neutrophil degranulation, but also increased cytokine secretion. Our results suggest that short‐term intensive fasting boost immune function, in particular innate immune function, at least in part by remodeling leukocytes expression profile.

## Graphical abstract

Short‐term intensive fasting remodels innate immunity in humans through improving neutrophil function with elevated secretion of cytokines, together with upregulation of autophagy and downregulation of apoptosis.

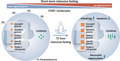

**Abbreviations**
BFS: breadth first search
DAG: directed acyclic graph
DEG: differentially expressed genes
DEP: differentially expressed proteins
DFS: depth first search
F.T.: Francisella tularensis
FDR: false‐discovery rate
GSEA: gene set enrichment analysis
PMA: para‐methoxyamphetamine
SigDEG(s): an intersection of unpaired and paired analysis differentially expressed gene(s)
sigDEP(s): an intersection of unpaired and paired analysis differentially expressed protein(s)

## 1 INTRODUCTION

Fasting has been considered an effective non‐medical intervention for fitness and delaying of aging agenda. Light or moderate fasting belongs to incomplete fasting format and mainly consists of intermittent fasting, fasting‐mimicking diets, and calorie restriction. The positive health benefits of incomplete fasting have been well documented (de Cabo & Mattson, 2019). Incomplete fasting was reported to reduce obesity (Bloom, 1959), cardiovascular diseases (Horne et al., 2008, 2013; Most et al., 2018), cancer (Ajona et al., 2020; Di Biase et al., 2016; Nencioni et al., 2018; Pietrocola et al., 2016; Yamaza et al., 2010), rheumatoid arthritis (Kjeldsen‐Kragh et al., 1991), metabolic syndrome (Steiniger et al., 2009), osteoarthritis (Schmidt et al., 2010), fibromyalgia(Michalsen et al., 2013), and improve memory (Witte et al., 2009). Through the use of animal fasting models, the mechanism of such health benefits has been becoming clear, such as regulation via ketogenesis (Capozzi et al., 2020; López‐Soldado et al., 2020; Steinhauser et al., 2018), hormone modulation (Fontana et al., 2016; Sutton et al., 2018; Volek et al., 2002), circadian clock (Gill et al., 2015), and gut microbiota changes that protect the central nervous system in autoimmunity disorders (Cignarella et al., 2018). Fasting also reduces inflammation (Jordan et al., 2019; Liang et al., 2021), promotes immunological memory (Collins et al., 2019), impacts immune cell dynamics and mucosal immune responses (Nagai et al., 2019), and increases stress resistance (Mladenovic Djordjevic et al., 2021), lipolysis (Kong et al., 2020), and autophagy(Liu et al., 2017).

In contrast to incomplete fasting, short‐term intensive fasting, such as beego, is complete water‐only fasting without regular food and minimal calories or supplements during the day and at night. Beego is a traditional fasting format for Chinese that lasts days to weeks initially developed for spiritual purpose and later extended to fitness. It involves psychological assistance such as meditation for Chinese, which is similar to Ramadan for introspection and prayer for Muslims. But beego is different from Ramadan since observers of Ramadan refrain from eating or drinking between sunrise and sunset each day by refeeding every night within an entire holy month (Sheikh & Wallia, 2007). The impact of Ramadan, which includes fasting on the day and refeeding at night during the holly month, on immunity remains controversial, with some reports showing benefits on the immune function, while others show no impact or harmful results (Adawi et al., 2017; Faris et al., 2012; Latifynia et al., 2009). Among other beneficial effects, Ramadan was found to attenuate inflammatory status by suppressing proinflammatory cytokine expression and decreasing circulating levels of leukocytes and body fat (Faris et al., 2012). Despite a decline in the neutrophil phagocytic and serum opsonization indexes, at the end of Ramadan, the percentage of neutrophils participating in phagocytosis increased with fasting. In addition, there was an increase in the percentage of neutrophils demonstrating nitro blue tetrazolium reduction. Therefore, Ramadan might have beneficial effects on neutrophil phagocytic function (Latifynia et al.,^2009^).

Unlike complete fasting, calorie restriction is one of the light or incomplete fasting formats. Short‐term calorie restriction does not result in beneficial effect on health. Early study shows that dietary restriction impairs neutrophil exudation by reducing CD11b/CD18 expression and chemokine production (Ikeda et al., 2001). In mice, long‐term (4‐months) calorie restriction influences innate and adaptive intestinal immunity. At the end of 4‐month fasting, lysozyme and phospholipase A2 gene levels were significantly increased, while IgA levels were diminished in the ileum due to reduced transport by its receptor pIgR. Additionally, long‐term calorie restriction differentially modified the expression of innate and adaptive immunity mediators in the intestine with increased gene expression of most cytokines (Suárez‐Souto et al., 2012). A recent study showed that short‐term (2–22 h) incomplete fasting in mice has differential transient effects on multiple organs (Huang et al., 2020). There, the authors identified T‐cell regulation as a central and prominent target of systemic modulations during short‐term fasting. Mechanistically, these changes seem to be driven by priming of adaptive immunity and the fine‐tuning of innate immune signaling. Similarly, a more recent study on intermittent fasting (overnight or 24‐h fasting followed by 3 h of refeeding) reports reduction in inflammation by blunting human CD4^+^ T helper cell activation and differentiation (Han et al., 2021).

More ongoing clinical trials are using fasting as a potential therapy, either alone or in combination with current front‐line treatments, for various health conditions (Longo et al., 2021). The TrueNorth Health Foundation has been conducting clinical study on water‐only fasting that shows largely positive outcomes with moderate adverse events (ClinicalTrials.gov, 2020a, 2020b, 2020c; Finnell et al., 2018). However, the biological effects of short‐term intensive fasting and the underlying mechanisms of such beneficial impact remain fundamentally unexplored. Our group recently performed a chronological study with beego participants who fasted for 7 or 14 days followed by a 7‐day programmed refeeding phase. In there, we observed that beego effectively decreased blood triacylglycerol (TG) selectively in TG‐high subjects, but not in TG‐normal subjects. Despite a transient cholesterol increase in all participants during fasting, these levels were normalized upon completion of the refeeding program. As a result, beego improved cardiovascular physiology and selectively reduced blood pressure in hypertensive subjects. Additionally, beego may coordinate and limit thrombosis risk by reducing platelet formation, activation, aggregation, and degranulation, while retaining normal hemostasis through sustained levels of coagulation factors and other hemostatic proteins (Fang et al., 2021). These studies suggest that, under medical supervision, beego can be implemented as a noninvasive intervention to reduce thrombosis risk. Since occasional short‐term intensive fasting is less dependent on professional supervision, this might prove easier to practice for fitness purposes as compared to long‐term intensive fasting. Here, we attempt to evaluate immune function after an occasional short‐term intensive fasting of 72 h.

## 2 RESULTS

### 2.1 Short‐term intensive fasting alters immunologic transcriptomic and proteomic profiles in leukocytes

**FIGURE 1:**
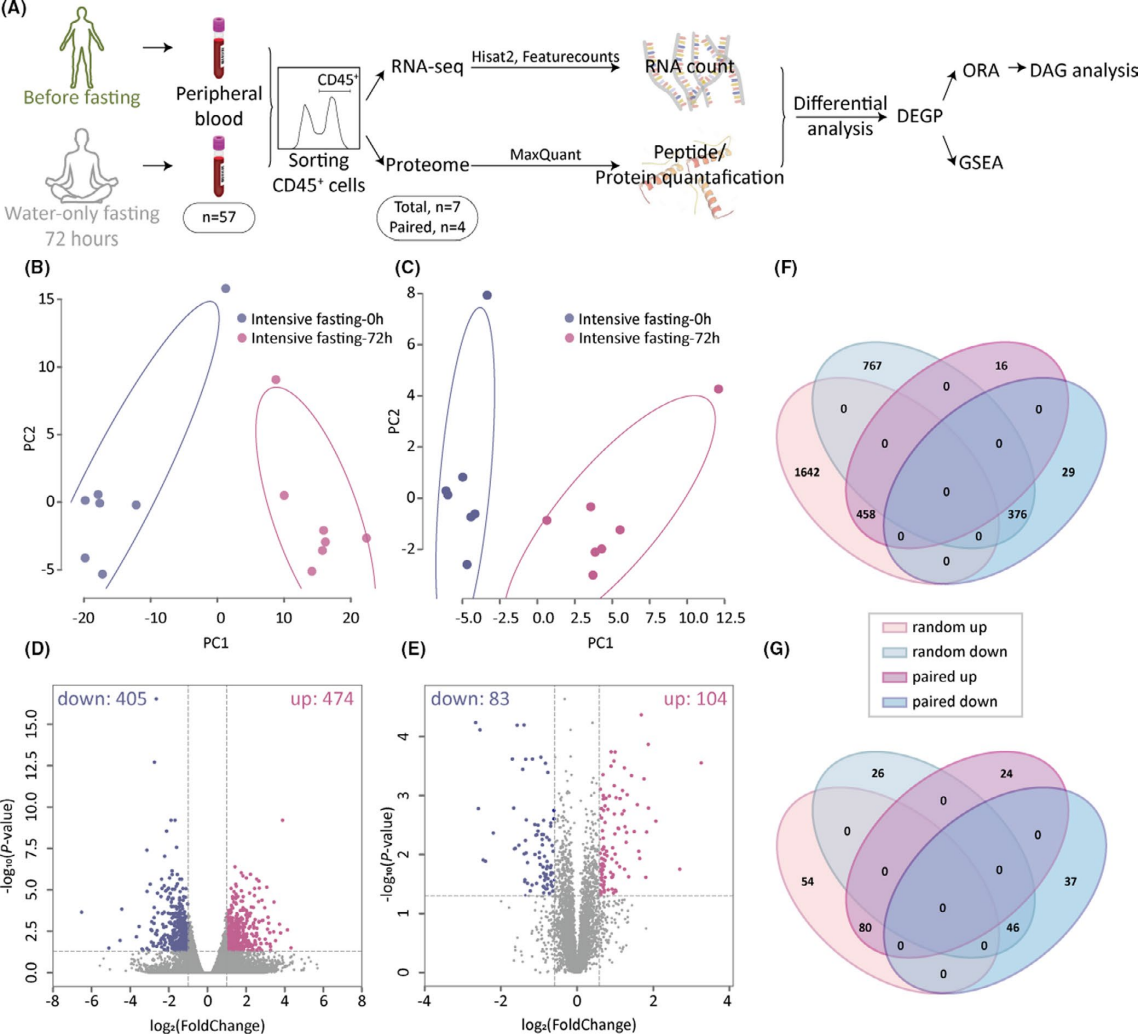
Short‐term intensive fasting alters the immune transcriptomic and proteomic profiles of leukocytes. (A) Schematic outline of intensive fasting regimes used in this study and experimental procedures conducted. (B) Two‐dimensional PCA of CD45^+^ cell transcriptome and (C) proteome from samples collected before and 3 days after intensive fasting. A total of 7 participants were included in this analysis. (paired *n* = 4, and unpaired *n* = 7). (D) Volcano plot of CD45^+^ cell transcriptome and (E) proteome. In the volcano plot, differentially expressed genes were determined under the condition of fold change ≥2 (proteins with fold change ≥1.5) and false‐discovery rate (adjusted *p* value) threshold ≤0.05. The negative values represent a decrease in gene expression and the positive values represent an increase in gene expression. The fold change was log‐transformed, and *p* value was transformed by −log10. Purple dots represent up‐regulated genes/proteins and blue dots represent down‐regulated genes/proteins. (F) Four‐way Venn diagram depicting significantly regulated genes and (G) proteins in CD45^+^ cells before and 72 h after intensive fasting. Significant molecular signatures are selected by intersection of the paired and unpaired analysis

To explore the impact of intensive fasting on innate immunity, we performed a multi‐omics analysis of circulating leukocytes from fasting subjects. Peripheral blood leukocytes (CD45^+^ cells) from 11 fasting participants were sorted and processed for transcriptomic and proteomic sequencing. The RNA and protein samples before and after 72 h of intensive fasting of four participants passed quality control, forming the paired analytical group. Also, in each sequencing experiment there are three additional samples at each time point that did not form sample pairs. RNA expression and protein abundance were quantified before and after 72 h of intensive fasting, and due to possible high variance in the gene expression profile across individual, the three additional unpaired samples were included in each analytical group for an unpaired analysis, which helped boost statistical power (Figure 1a). Principal component analysis of all samples’ transcriptomic (Figure 1b) and proteomic profiling (Figure 1c) exhibited distinct signatures between the two time points, intensive fasting 0 h (on the morning just before the fasting begins) and intensive fasting 72 h (on the morning just after 72‐h fasting finishes). A total of 37, 963 mRNAs were identified in paired transcriptomic profiling (*n* = 4). Unpaired analysis (*n* = 7) showed 3, 243 differentially expressed genes (DEGs), in which 2, 100 genes were up‐regulated and 1, 143 genes were down‐regulated. Paired analysis produced a total of 879 DEGs, with 474 genes up‐regulated and 405 genes down‐regulated (Figure 1d). Therefore, the intersection of the DEGs from the two analytical schemes was defined as the significant DEG set in response to fasting, where 458 genes were up‐regulated and 376 genes were down‐regulated (Figure 1f). Proteomic profiling yielded a total of 4, 839 proteins, with the paired differential expressed proteins (DEPs) set containing 104 up‐regulated proteins and 83 down‐regulated proteins (Figure 1e). The significant DEP set in response to fasting was composed of 80 up‐regulated and 46 proteins down‐regulated proteins (Figure 1g).

### 2.2 Functional enrichment on DEGs and DEPs reveals pathways critical to immune cell functions

**FIGURE 2:**
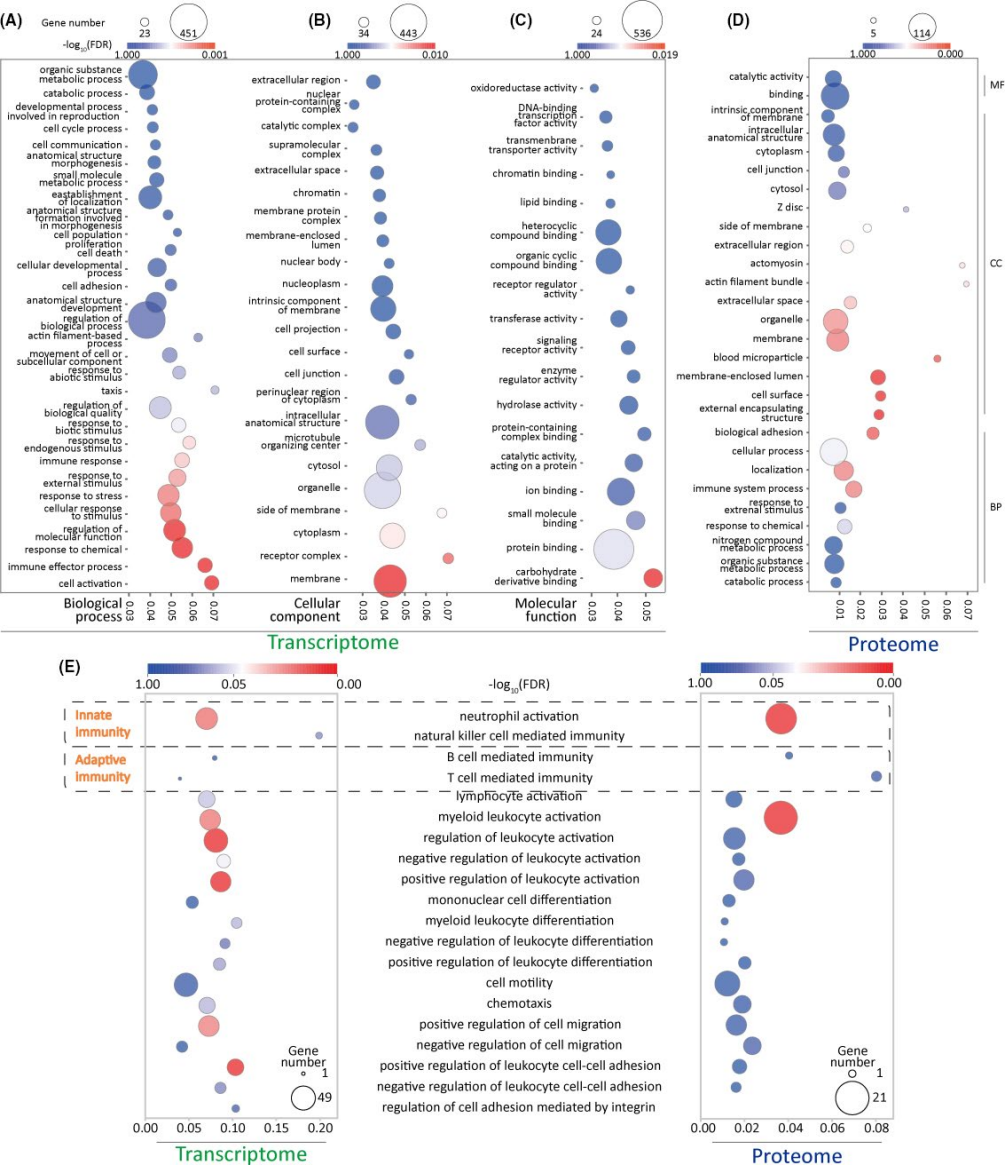
Multi‐omics enrichment analysis of leukocyte transcriptome and proteome reveal activated metabolic processes and shifted immunologic functions after short‐term intensive fasting. Gene ontology enrichment analysis on (A) biological process (BP), (B) cellular component (CC), and (C) molecular function (MF) of transcriptomic data. (D) Gene ontology enrichment analysis on BP, CC, and MF of proteomic data. Dot size represents the number of genes in each pathway, and the color ranging from red to blue corresponds to statistical significance, false‐discovery rate (FDR), from most significant to least significant. (E) Bubble plot depicting DEGs (left), and DEPs (right) associated with leukocyte functions. Color bar represents the FDR of each enriched pathway, ranging from low to high, in correspondence red to blue

To determine biological pathways that are activated or silenced during the intensive fasting process, we employed overrepresentation analysis on both our omics data using GOAtools, a python‐based library (Klopfenstein et al., 2018). Pathways with either a false‐discovery rate (FDR) lower than 0.05 or study count higher than 20 (15 in proteomics) were considered as biological insightful. The enrichment analysis on sigDEGs produced 549 pathways in gene ontology biological process (GOBP), 74 in cellular component (GOCC), and 101 in molecular function (GOMF). Enrichment on sigDEPs produced 155 enriched pathways in GOBP, 77 in GOCC, and 15 in GOMF. Gene ontology database has been known for its hierarchal structure, so we next employed both breadth first search (BFS) and depth first search (DFS) to interpret the enrichment results. We used BFS to summarize the key molecular changes after 72 h of intensive fasting. As shown in Figure 2a–d, intensive fasting clearly influences immunological functions, immune response, metabolic process, and response to stimulus. DFS analysis revealed a distinct enrichment in leukocyte‐mediated immune functions, particularly processes related to innate immunity. Further analysis revealed other aspects involved in leukocyte functions, including cell motility and chemotaxis, which are both integral functions in leukocyte activation (Figure 2e).

### 2.3 Intensive fasting enhances autophagy level of leukocytes

**FIGURE 3:**
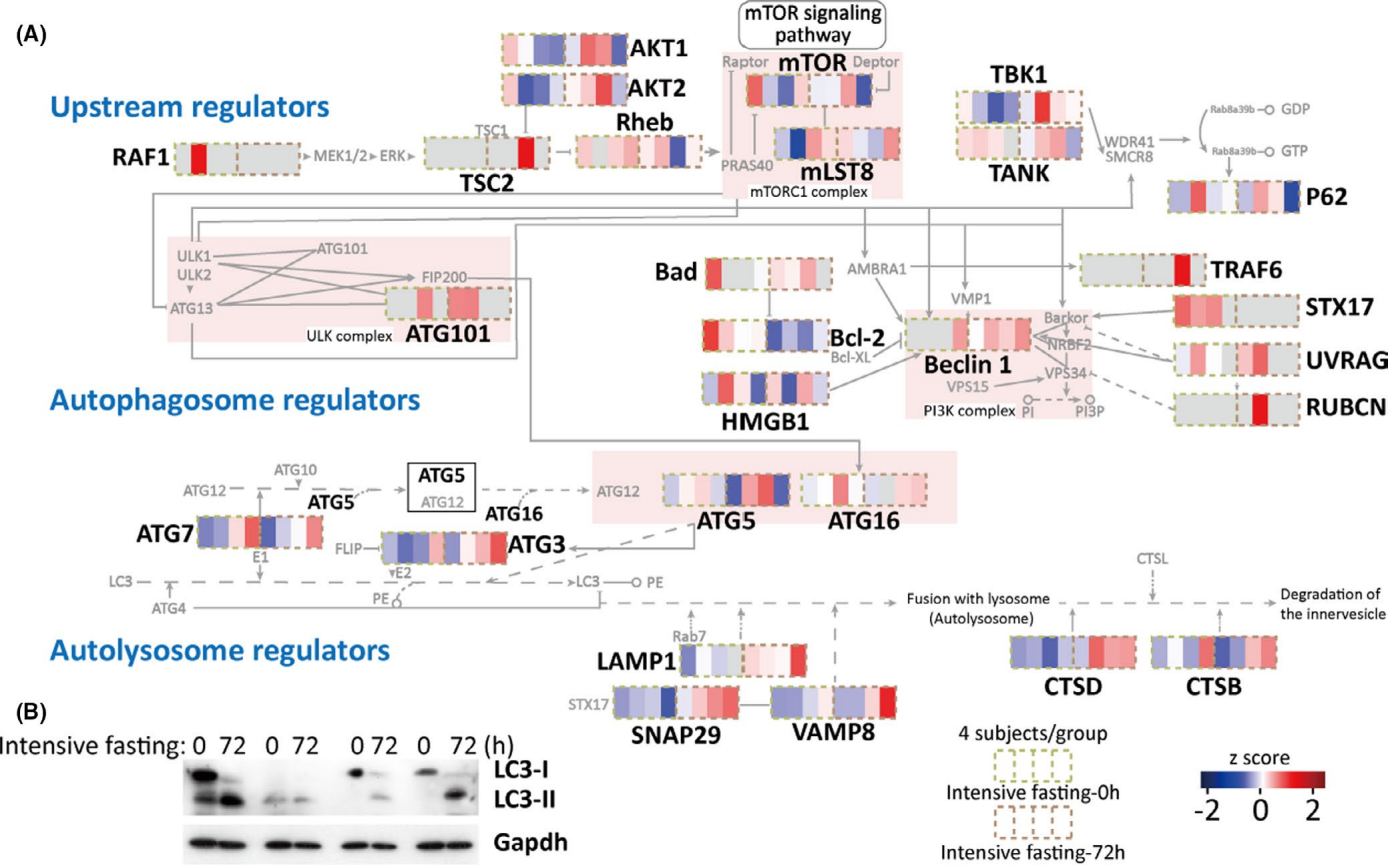
Intensive fasting increases autophagy level of leukocytes. (A) Annotated heatmap of differentially expressed proteins involved in the autophagy process. The signaling pathway is adapted from kegg pathway hsa04140. (B) Measurement of LC3 protein by western blotting

Autophagy is a metabolic mechanism which safeguards cell homeostasis by lysosomal degradation of aged or damaged organelles and proteins aggregates, thereby facilitating bioenergetic homeostasis. Starvation is known to trigger autophagy in cell lines and animal models, but in vivo characterization of autophagy activity in human body remains challenging since autophagic flux assays require treatment with pharmacological inhibitors, which is toxic. Thus, high‐throughput sequencing provides a feasible way to address this question. Our proteomic profiling revealed that a group of mammalian autophagy‐related proteins including major autophagy machinery members (Beclin1, Atg3, Atg5, Atg16, etc.), autolysosome members (LAMP, SNAP29, VAMP28, CTSD, CTSB, etc.), and autophagy upstream regulators (TSC2, Akt, TBK1, etc.) were up‐regulated during intensive fasting. Moreover, proteins known to inhibit autophagy such as Bcl‐2 are generally down‐regulated (Figure 3a).

To validate these results, we assess LC3 expression, a key indicator for the formation of autophagosome, by immunoblotting assay, and show that the ratio between LC3‐II/LC3‐I was significantly elevated 72 h after intensive fasting (Figure 3b), further suggesting that intensive fasting increase leukocyte autophagy activity, which helps maintain a better cell homeostasis.

### 2.4 Intensive fasting reduces apoptosis level of leukocytes and increased neutrophils

**FIGURE 4:**
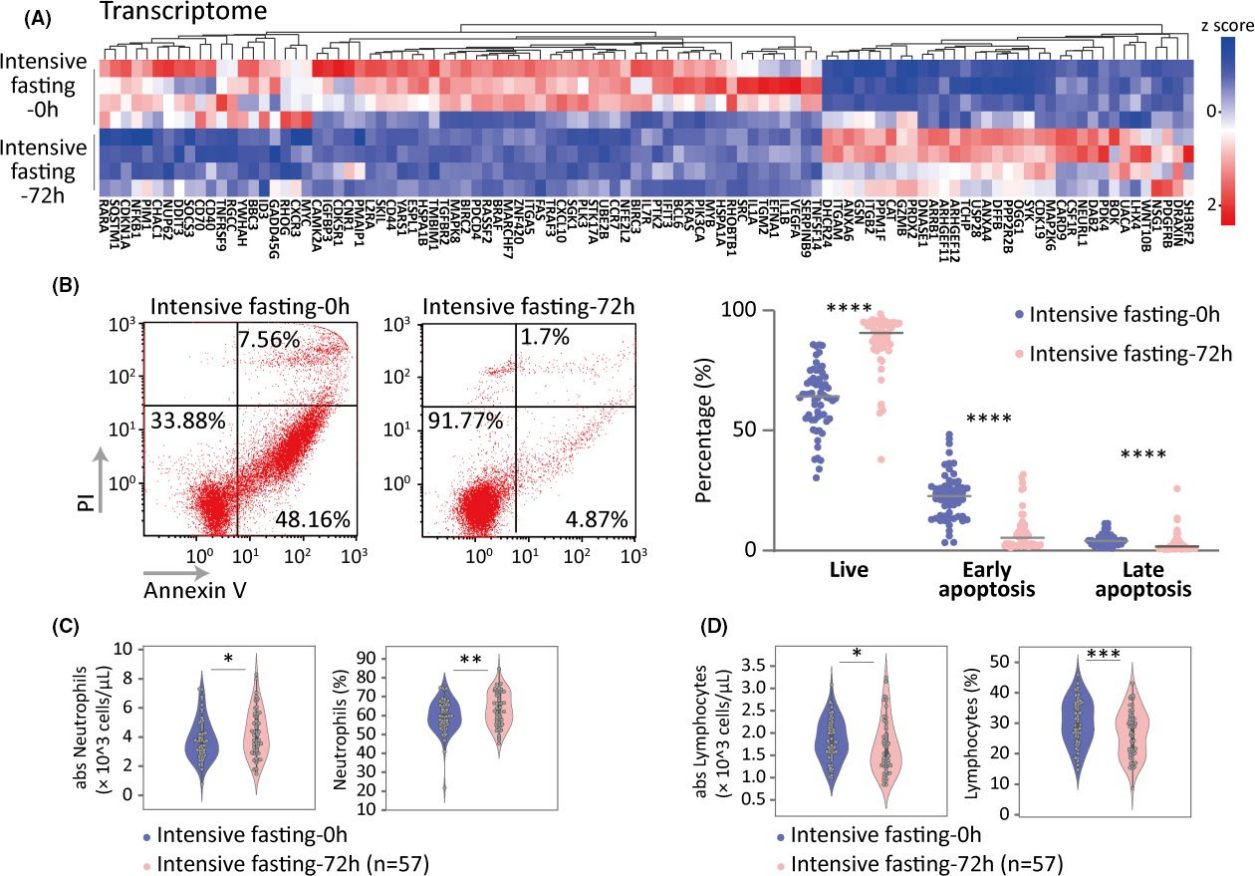
Intensive fasting reduces apoptosis of leukocytes. (A) DEGs enriched in the apoptotic and regulation of apoptotic process. All pathways are statistically significant enrichment. (B) Representative flow cytometry plot for measurement of apoptotic CD45^+^ cells before and 72 h after intensive fasting (left panel). Quantification of the flow cytometry on apoptosis of CD45^+^ population (right panel). Wilcoxon matched‐pairs signed rank test is used. *p value <0.05, **p value <0.01, ***p value <0.001, ****p value <0.0001. (C, D) Peripheral neutrophils and lymphocytes count at before (Control) and 72 h after intensive fasting (last day of the fasting)

Physiological autophagy is a protective mechanism to protect cells during starvation and other stresses, and its activation often inhibits apoptosis. Transcriptomic profiling revealed a majority of down‐regulated genes related to apoptotic activity (69 out of 104 sigDEGs). This suggests that intensive fasting lowers apoptosis activity of leukocytes (Figure 4a). Measurement of apoptotic status by flow cytometry analysis showed a significant increase in the percentage of live cells (Annexin V and PI double negative) in CD45^+^ leukocyte population from 57 subjects at 72 h after intensive fasting, confirming reduced apoptosis of leukocytes by intensive fasting (Figure 4b).

To explore the response to fasting in various leukocytes populations, we examined the peripheral leukocyte counts from 57 participants after intensive fasting. Statistical analysis on complete blood count data revealed major shifts in leukocyte composition. Noticeably, neutrophil showed significant elevation in both total cell number and frequency, which was associated with a reduction in lymphocytes, but within its normal value range (Figure 4c,d), suggesting that intensive fasting favors innate immune enhancement.

### 2.5 Neutrophil activation is the main immunological response to intensive fasting

**FIGURE 5:**
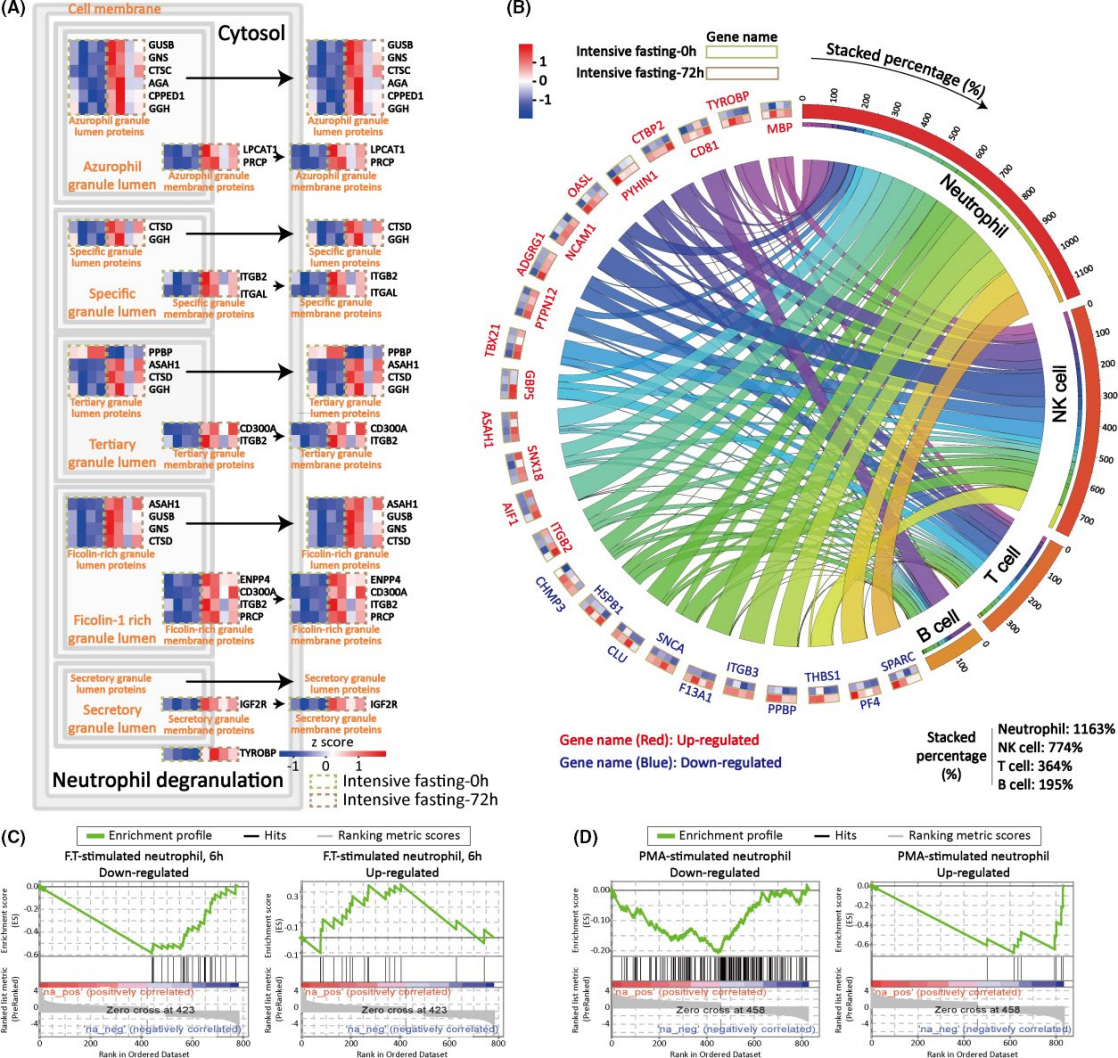
Neutrophil activation is the key immunological change in response to intensive fasting. (A) Heatmap of proteomic profiling on neutrophil degranulation. Expression data were row normalized to illustrate the differential status. (B) Circos plot depicting DEPs with cytokines activity and their origins annotated by HPA database. Total value for each cytokines sums up to 100% and total value for each cell type is stacked percentage from their corresponding cytokines. Heatmap illustrating the relative expression at intensive fasting 0 and 72 h is plotted around circos plot. The outer block corresponds to expression at 0‐h intensive fasting, and the inner block corresponds to expression at 72‐h fasting. Expression data are normalized for each protein. (C, D) GSEA analysis of dataset from PMA‐ and F.T.‐ stimulated neutrophils

Since CD45^+^ population comprises mainly neutrophils, macrophages/monocytes, and lymphocytes, we next identified the molecular signatures specific to each cell lineage. Significantly more DEGs/DEPs associated with neutrophil degranulation were identified in the enrichment of descendants of leukocyte‐mediated immunity. Neutrophils are largely responsible for pathogen clearance through phagocytosis, degranulation, and the release of neutrophil extracellular traps, but can also modulate the immune response by interacting with other immune cells such as antigen‐presenting cells or lymphocytes (Kanashiro et al., 2020). Both transcriptomic and proteomic profiling revealed significantly increased enrichment in neutrophil degranulation (Figure 2e, Figure S1), suggesting activation of neutrophils after intensive fasting. All proteins involved in neutrophil degranulation showed significant upregulation, except for the pro‐platelet basic protein, PPBP (Figure 5a). In contrast, no detectable changes were found in phagocytosis and neutrophil extracellular traps (NETs).

Cytokines released from innate immune cells plays a key role in the regulation of the immune response. These signaling molecules initiate or constrain inflammatory responses to infection and injury (Lacy & Stow, 2011). Notable changes were detected in the expression of cytokines in various leukocytes in response to intensive fasting. Figure 5b illustrates the contribution of neutrophils, NK cells, T cells, and B cells to cytokine secretion. Specifically, NCAM1, HSPB1, F13A1, and PF4 varied significantly in expression level among participants before intensive fasting but stabilized 72 h after the fasting (Figure S2). Neutrophil and natural killer cells were the main drivers of cytokine responses as manifested by stacked percentages 1163% and 774%, respectively, for neutrophil and natural killer cells (Figure 5b). Next, we used gene set enrichment analysis (GSEA) on an immunological signature database (Liberzon et al., 2015) to verify these results. GSEA analysis of the transcriptomic profiles showed mainly positive correlation between intensive fasting and stimulated neutrophils. Particularly, one dataset from neutrophils treated with bacterium Francisella tularensis (GSE37416, Schwartz et al., 2013) showed positive correlated expression pattern (Figure 5c). Similarly, another dataset (GSE126757) from a commonly used neutrophil stimuli, para‐methoxyamphetamine (PMA), was also considered in verifying the stimulated status of CD45^+^ population after intensive fasting, and again showed positive correlation (Figure 5d). Given the elevated neutrophil count and frequency 72 h after intensive fasting, we conclude that intensive fasting works as a stimulus in neutrophil activation.

To evaluate a broader impact of intensive fasting on immune response, we performed biochemical analysis on 57 fasting subjects, which showed elevated levels of serum total protein, serum albumin, and serum globulin, but within the normal range. Serum albumin/globulin ratio remained unchanged while serum pre‐albumin decreased (Figure S3), suggesting that intensive fasting strengthen overall immunity by promoting serum protein production.

## 3 DISCUSSION

**FIGURE 6:**
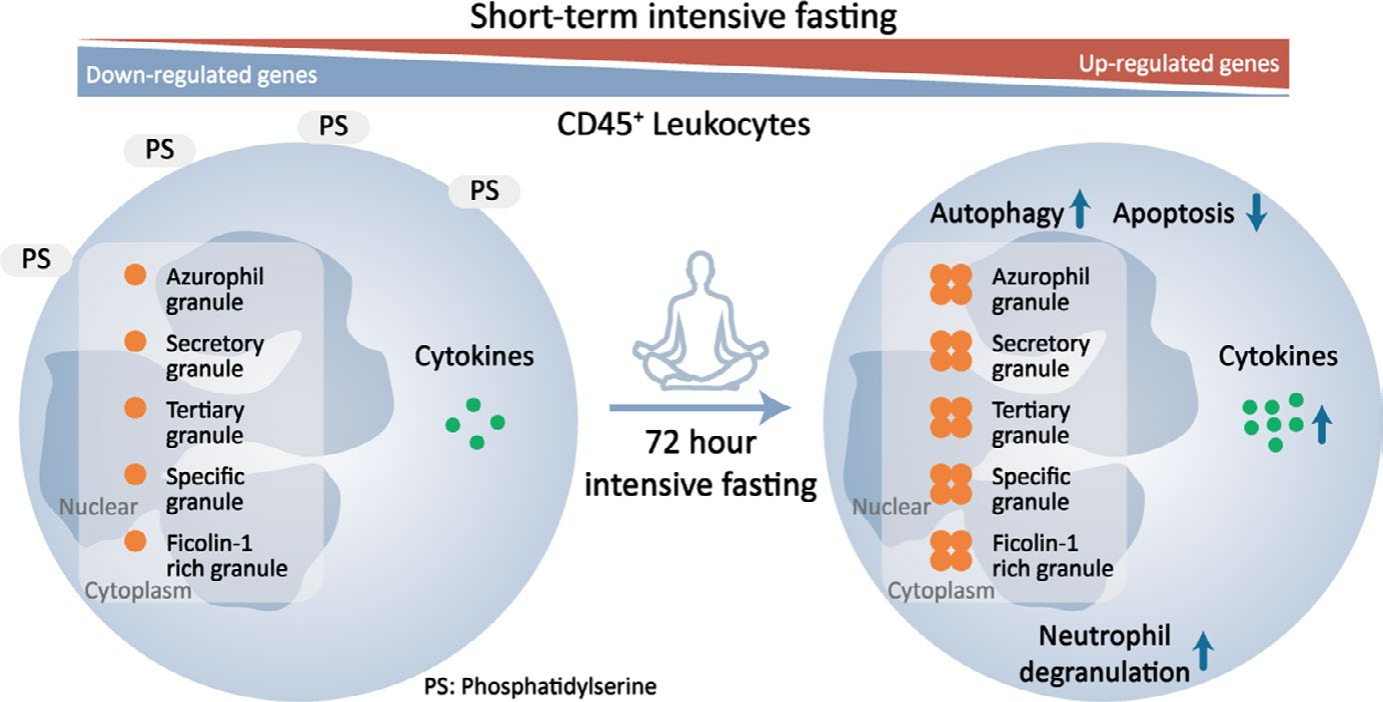
Short‐term intensive fasting remodels innate immune function in humans. 72‐h intensive fasting improves neutrophil function with elevated secretion of cytokines. The fasting‐triggered upregulation of autophagy and downregulation of apoptosis together contribute to the improved innate immunity

In this clinical study, we applied multi‐omics tools to examine the impact of short‐term intensive fasting on immune function. Our analysis suggests that occasional short‐term intensive fasting enhances immune function, particularly, neutrophil activity. Presumably, this is achieved at least in part by remodeling of the leukocyte's expression profiles (Figure 6).

Unlike previous work which primarily focuses on circulating peripheral blood mononuclear cells and lymphocytes (Han et al., 2021; Huang et al., 2020), here we sorted CD45^+^ leukocytes cells to investigate both the differentials in innate and adaptive immune system. CD45^+^ cells are composed of myeloid, natural killer cells, T cells, B cells, and plasma cells. The ratios of lymphoid and myeloid cells are relatively close in the CD45^+^ population (Zhao et al., 2020), enabling us to investigate the biological changes in human immune system. Neutrophils are generally the first responders to bacterial infection and play a pivotal role in initiation and mediation of immune response. Neutrophils exercise their bactericidal ability via a series of biological processes including phagocytosis, secretion of proteases, production of reactive oxygen species, and release of neutrophil extracellular traps (Manfredi et al., 2018; Nathan, 2006). Our transcriptomic and proteomic analysis of CD45^+^ cells revealed activated neutrophil function and neutrophil degranulation process, accompanied by increased granule contents, which suggests increased granule production. This degranulation process is a regulated exocytosis of secretory granules containing mediators such as proteases, lipases, and inflammatory mediators. Other functions related to neutrophil activation, such as cell migration and cell adhesion, also displayed positive regulation in response to intensive fasting.

The activation profile of neutrophils 72 h after intensive fasting resembled that of chemical (PMA) and biological (F. Tularensis) stimulation. PMA has been widely used to induce neutrophil activation in vitro, inducing the release of superoxide anion and neutrophil extracellular traps (Kenny et al., 2017). Similarly, F. Tularensis, a pathogenic species of Gram‐negative coccobacillus, has been reported to not only activate neutrophil‐mediated immunity, but also to be closely related to longer neutrophil lifespan and subsequently prolonged inflammation. Comparative GSEA on transcriptomic profiling with PMA‐stimulated neutrophils (GSE126757) and F. Tularensis stimulated neutrophils showed molecular variations similar to those observed here at the end of 72‐h intensive fasting. In the process of neutrophil activation, integrin family protein showed variation in both transcripts and protein levels, which have been closely related to leukocyte‐mediated immune activation. Particularly, integrin ITGAL/ITGB2, accompanied with ICAM3, has been reported to contribute to neutrophil efferocytosis of macrophages (Kristóf et al., 2013).

Cathepsin family contains a series of lysosomal proteases that have been implicated in a wide variety of inflammatory activities. Cathepsin C (CTSC) is known to function as the key activator of elastase, and cathepsin G (CTSG) in neutrophils has been reported as a pathogenesis signature in many auto‐immune and chronic inflammatory diseases (Burgener et al., 2019), whereas cathepsin D (CTSD) has been related not only to immune responses but also regulation of programmed cell death. Considering these factors that weigh greatly in the immune response, these data further suggest that occasional intensive fasting can act as a trigger of the innate immune response. On the contrary, cytokines related to platelet activation are decreased. This is consistent with our recent report on the reduction of blood clots risk by beego (Fang et al., 2021).

A previous report showed that overnight or 24‐h fasting can blunt CD4^+^ Th cells activation and differentiation, which may be exploited as an intervention on auto‐immune diseases (Han et al., 2021). In this intensive fasting model, we also discovered from transcriptomic profiling on leukocytes that genes involved in lymphocyte differentiation are prone to be negative regulated during intensive fasting. However, peripheral blood count indicates that participants had statistically lower lymphocytes, but within normal range after 72‐h intensive fasting. Thus, our results suggest that intensive fasting favors strengthening of innate immunity.

Autophagy is implicated in multiple functions of various tissues, including but not limited to decelerating of aging and suppression of oncogenesis (Cao et al., 2015; Fang et al., 2020; Yuan et al., 2020). In vivo autophagy is effectively activated in response to intensive fasting (Figure 3a,b). This presumably leads to digestion of aged proteins/organelles into amino acids and free fatty acids for recycling and regeneration of new proteins and organelles. Autophagy initiation is highly dependent on Beclin1, a key member in the Beclin1‐PI3K C3 complex (Kroemer et al., 2010). Beclin1 upregulation enhances autophagy (Wang et al., 2009). Interestingly, different formats of fasting impacts autophagy in a slightly different way in the regulation of p62, which was reduced in response to intensive fasting in our study (Figure 3a), but was increased in the incomplete fasting regimes (Bagherniya et al., 2018). On the contrary, the apoptotic process in leukocytes is prone to suppression after 72‐h intensive fasting, which is proven by flow cytometry and omics signatures (Figure 4). This is in consistence with the role of Beclin1 that activates autophagy but inhibits apoptosis (Wang, 2008). Interestingly, short‐term intensive fasting enhances immune function, but does not aggravate prolonged inflammation which could cause subsequent harm to those with chronic inflammation. In our recent study, we monitored the hematological profile seven days and in some participants 14 days of intensive fasting after 7‐day refeeding process, and the composition of their leukocyte's populations were more balanced after intensive fasting (Fang et al., 2021). Nevertheless, it would be helpful to examine the short‐term and long‐term effects of occasional intensive fasting on other clinical complications such as chronic inflammation or tissue damage.

Many viruses prohibit host autophagy by blocking autophagy‐triggered pathways. A recent study has indicated that severe acute respiratory syndrome coronavirus 2 (SARS‐CoV‐2) also inhibits host autophagy activity and in vitro administration of spermidine, AKT inhibitor MK‐2206, and the Beclin‐1 stabilizing, anti‐helminthic drug niclosamide effectively inhibits SARS‐CoV‐2 propagation (Gassen et al., 2020), suggesting a potential application of autophagy against SARS‐CoV‐2 infection. Compromised immunity has a high risk of viral disease. Due to quick mutations of fatal viruses such as coronavirus disease‐19 (COVID‐19) that challenge the effectiveness of timely developed drugs and vaccines, strengthening immunity through both medical and non‐medical interventions, including but not limited to fasting, may be helpful for surviving this disease (Bartleson et al., 2021; Hannan et al., 2020). Intensive fasting causes an extreme starvation stress, which presumably triggers higher activation of autophagy than incomplete fasting does, possibly leading to a better priming of the host immune system to fight against virus diseases.

However, our trial has several limitations. It is difficult to recruit a comparable control group for sampling since the subjects in this fasting study have different subhealthy conditions. It is also not easy to organize large cohort in the same location for several days eating nothing but being requested to donate blood samples. The quantity for each sampling was fully based on the informed consent from the volunteered subjects. The quality of the samples also varied among subjects and even differed in the same subject between two sampling times (0‐ and 72‐h fasting), leading to small size of cohort for multi‐omics analysis and some samples are unpaired. However, the number of subjects used for laboratory biochemical analysis is much bigger since much less quantity for each sample was required.

Psychological induction is known to have an influence on immune function (Black & Slavich, 2016; Davidson et al., 2003). Validation with laboratory animals for psychological influence on intensive fasting is virtually impossible. We noticed that laboratory mice could not survive 4‐day water‐only fasting. In addition, there was a big variance in the age of the subjects used for our analysis and we were unable to group the data within a similar age although we realized that the immune system undergoes age‐associated changes. Future study with age‐grouped larger cohort is warranted to deepen our understanding of the impact of intensive fasting on immune system and establish direct link between psychological intervene and fasting effect.

Finally, this study focused on leukocytes expression profiling in the context of intensive fasting. Post‐translational modification of proteins and epigenetic modulation of the genome and macromolecules may also be implicated in reshaping immune function in response to intensive fasting. Therefore, we recognize that changes in gene expression patterns at transcriptional and translational levels are only partially responsible for innate immune remodeling by intensive fasting.

## 4 CONCLUSION

We observed that occasional short‐term intensive fasting has a significant effect on immune function, especially innate immunity. Leukocyte survival can be promoted by enhanced autophagy and decreased apoptosis 72 h after intensive fasting. The degranulation of neutrophils increased significantly, and cytokines that govern immune cells are mostly increased, largely released by neutrophils. This suggests that occasional intensive fasting enhances innate immune function. In summary, our study provides a comprehensive transcriptomic and proteomic profiling of leukocytes after 72‐h intensive fasting and provides new insights into the pathways involved in the systemic immune remodeling after occasional short‐term intensive fasting. Altogether, this suggests that occasional short‐term intensive fasting may be exploited as an immune‐modulatory intervention.

## 5 EXPERIMENTAL PROCEDURES

### 5.1 Ethics

The study was approved from Institutional Ethics Review Board at Soochow University (Approval No. ECSU‐2019000153) and the Chinese Clinical Trial Register, an official review board of China for clinical trial (Registration No. ChiCTR1900027451). 11 participants aged from 29 to 60 years old for the multi‐omics study (Table S1), 57 participants aged from 16 to 59 for blood routine test (Table S2), and 40 participants aged from 27 to 67 for biochemical analysis (Table S3; statistic data presented solely in Figures S3; were from the same subjects in an earlier fasting event), and were recruited by the research team. Participants were enrolled after giving their written informed consent.

### 5.2 Fasting protocol

Intensive fasting was done in the way as described earlier (Fang et al., 2021). To facilitate the intensive fasting, psychological induction such as meditation was conducted with the subjects during the fasting period. The sampling used for this study was taken at 0 and 72 h of the fasting to examine the impact on immune response from a short‐term intensive fasting. The participants chosen for this study were solely based on their informed consent on providing more blood sampling sufficient for multi‐omics study.

### 5.3 Flow cytometry cell sorting

Peripheral blood mononuclear cells were harvested by density centrifugation (Ficoll‐Paque, GE Healthcare Life Sciences). Cells were stained with fluorochrome‐conjugated antibody (CD45 FITC, eBioscience), and CD45‐positive cells were sorted by flow cytometry (BD FACSAria III). Cells were stained with antibody (CD45 BV421, Biolegend) and analyzed apoptosis by kit (FITC annexin V apoptosis detection kit, BD).

### 5.4 mRNA library construction

Total RNA was extracted from the tissues using Trizol (Invitrogen) according to manual instruction. Oligo(dT)‐attached magnetic beads were used to purify mRNA. The purified mRNA was then fragmented into small pieces with fragment buffer at appropriate temperature. First‐strand cDNA was generated using random hexamer‐primed reverse transcription, followed by a second‐strand cDNA synthesis. A‐Tailing Mix and RNA Index Adapters were then added by incubating. The cDNA fragments obtained from previous step were amplified by PCR, and products were purified by Ampure XP Beads and then dissolved in EB solution. The product was validated on the Agilent Technologies 2100 bioanalyzer for quality control. The double‐stranded PCR products from previous step were heated denatured and circularized by the splint oligo sequence to get the final library. The single‐strand circle DNA was formatted as the final library. The final library was amplified with phi29 to make DNA nanoball (DNB) which had more than 300 copies of one molecular, DNBs were loaded into the patterned nanoarray and single end 50 bases reads were generated on BGIseq500 platform (BGI).

### 5.5 Reference genome and annotation

Human genome sequence (primary assembly, GRCh38.p13) fasta file, comprehensive gene annotation GTF file (primary assembly, GENCODE release 36), and metadata (UniProtKB/SwissProt entry associated with the transcript, GENCODE release 36) were downloaded from GENCODE website. The index for HISAT2 was built using GENCODE genome sequences according to hisat2‐build manual. Gene features (gene length, gene‐transcript‐swissprot‐id conversion) were generated from the GTF file using in‐house scripts.

### 5.6 mRNA quantification pipeline

All 14 sequenced fastq files were generated and cleaned by BGI; then, we aligned the RNA sequencing reads using HISAT2 (version 2.2.1) (Kim et al., 2019) with self‐built ht2 index (Alignment result is listed in Table S4). The parameters were default for single‐end analysis. Aligned reads were stored in SAM files. Next, we sorted SAM files into BAM files as well as for compression using SAMtools(version 1.11) (Danecek et al., 2021). For quantification, featureCounts (Liao et al., 2014) from Subread package (version 2.0.1) was used with comprehensive gene annotation GTF with exon as feature type and with gene id as meta feature. Quantification results were stored in tab‐delimited format.

### 5.7 Principal component analysis

Two‐dimensional principal component analysis (PCA) was conducted using python library Scikit‐learn module PCA (Pedregosa et al., 2011). RNA read count matrix was normalized using sklearn.preprocessing. PowerTransformer class. Protein abundance was normalized using *z*‐scoring. PCA scatter plots were plotted using python library Seaborn (Ref https://doi.org/10.21105/joss.03021). Confidence ellipse was also generated with two standard deviations to the corresponding group values.

### 5.8 RNA‐Seq differential expression analysis

DEG analysis was performed using R package DESeq2 (version 1.30.0) (Love et al., 2014). Paired analysis was performed with the condition parameter included with both individual identities for each sample and treatment, while unpaired analysis was performed with only the treatment parameter. We determine DEGs with |log_2_FoldChange| >1 and adjusted *p* value <0.05. Venn diagram was plotted using python library matplotlib with up/down DEGs from those two analytical approaches. A intersect set of DEGs (SigDEGs) of paired DEGs and unpaired DEGs were extracted for further analysis.

### 5.9 Proteomic extraction

Leukocyte sample was sonicated three times on ice using a high‐intensity ultrasonic processor (Scientz) in lysis buffer (8 M urea, 1% Protease Inhibitor Cocktail). (Note: For PTM experiments, inhibitors were also added to the lysis buffer, for example, 3 μM TSA and 50 mM NAM for acetylation) The remaining debris was removed by centrifugation at 12,000 g at 4°C for 10 min. Finally, the supernatant was collected and the protein concentration was determined with BCA kit according to the manufacturer's instructions.

### 5.10 Trypsin digestion

For digestion, the protein solution was reduced with 5 mM dithiothreitol for 30 min at 56°C and alkylated with 11 mM iodoacetamide for 15 min at room temperature in darkness. The protein sample was then diluted by adding 100 mM TEAB to urea concentration <2 M. Finally, trypsin was added at 1:50 trypsin‐to‐protein mass ratio for the first digestion overnight and 1:100 trypsin‐to‐protein mass ratio for a second 4 h‐digestion.

### 5.11 LC‐MS/MS analysis

The tryptic peptides were dissolved in 0.1% formic acid (solvent A), directly loaded onto a home‐made reversed‐phase analytical column. The gradient was comprised of an increase from 6% to 23% solvent B (0.1% formic acid in 98% acetonitrile) over 26 min, 23% to 35% in 8 min and climbing to 80% in 3 min then holding at 80% for the last 3 min, all at a constant flow rate of 400 nl/min on an EASY‐nLC 1000 UPLC system. The peptides were subjected to NSI source followed by tandem mass spectrometry (MS/MS) in Q ExactiveTM Plus (Thermo) coupled online to the UPLC. The electrospray voltage applied was 2.0 kV. The *m*/*z* scan range was 350–1800 for full scan, and intact peptides were detected in the Orbitrap at a resolution of 70,000. Peptides were then selected for MS/MS using NCE setting as 28 and the fragments were detected in the Orbitrap at a resolution of 17,500. A data‐dependent procedure that alternated between one MS scan followed by 20 MS/MS scans was applied with 15.0s dynamic exclusion. Automatic gain control (AGC) was set at 5E4. Fixed first mass was set as 100 *m*/*z*.

### 5.12 Protein database search

The resulting MS/MS data were processed using Maxquant search engine (v.1.5.2.8). Tandem mass spectra were searched against human uniprot database concatenated with reverse decoy database. Trypsin/P was specified as cleavage enzyme allowing up to four missing cleavages. The mass tolerance for precursor ions was set as 20 ppm in First search and 5 ppm in Main search, and the mass tolerance for fragment ions was set as 0.02 Da. Carbamidomethyl on Cys was specified as fixed modification and acetylation modification and oxidation on Met were specified as variable modifications. False‐discovery rate (FDR) was adjusted to <1% and minimum score for modified peptides was set >40.

### 5.13 Differential protein expression analysis

We first calculated the relative expression based on samples’ LFQ intensity. Protein expression differences are determined based on the ratio of Fasted versus Control. Statistical significance is determined using two‐tailed Student's *t* test. We determine DEPs with |log2FoldChange| >log2(1.5) and adjusted *p* value <0.05.

### 5.14 Gene ontology and annotations

Gene ontology OBO file (release 1 February 2021) was downloaded from geneontology.org. Human gene ontology annotation GAF file (release 1 December 2020) was downloaded from EBI Gene Ontology Annotation Database.

### 5.15 Gene ontology enrichment analysis

We conducted overrepresentation analysis on DEGs/DEPs identified from differential analysis using python library GOAtools. All DEG enrichment query lists were converted from Ensemble gene id to protein id for convenience. Enrichment background was generated from gene ontology annotation GAF file using in‐house script. Criterion for identifying significantly overrepresented terms was set as adjusted *p* value <0.05.

### 5.16 Gene ontology DAG search

Ancestors and descendants of certain pathways were identified using in‐house directed acyclic graph (DAG) search module derived from GOAtools. All search results were validated manually.

### 5.17 Gene set enrichment analysis

We conducted Gene Set Enrichment Analysis (GSEA) on pre‐ranked gene lists with log_2_FoldChange as ranking metric using GSEA v4.1.0 (Subramanian et al., 2005). We used gene ontology biological process terms as gene sets, and the gene sets were generated using in‐house script from gene ontology associations middleware from gene ontology analysis. We use nominal *p* value with the criteria of nominal *p* value lower than 0.05 to identify significant GSEA results.

### 5.18 Cytokine contribution analysis

Expression data were extracted from Human Protein Atlas. Original expression metric was normalized for each cytokine to indicate the contribution of each type, and the percentages of all cytokines were stacked in each cell type to illustrate the overall contribution from each cell type.

### 5.19 Human peripheral blood Acquisition and laboratory biochemical analysis

The blood is mixed promptly with the EDTA to avoid sample clotting. The samples were analyzed on the automatic five categories hematology analyzer (Siemens). Blood routine test and laboratory biochemical analysis were performed in the way described previously (Fang et al., 2021). Briefly, whole blood without anticoagulant was incubated at 4°C overnight and then centrifuged at 500 g for 20 min at room temperature. All serum samples were separated and frozen at −80°C until laboratory testing. Serum was analyzed for blood lipids with the Chemistry Analyzer (Roche, cobas c702) by Dian Diagnostics, China. This information has been included in the revised manuscript.

### 5.20 Statistical analysis

Absolute values in complete blood count were analyzed using paired Student's *t* test, and percentages were analyzed using Wilcoxon matched‐pairs signed rank test. Percentages of apoptotic status in flow cytometry were analyzed using Wilcoxon matched‐pairs signed rank test.

## CONFLICT OF INTEREST

All authors declare no competing interests.

## AUTHOR CONTRIBUTIONS

JQ and YF designed the study, analyzed the data and wrote the manuscript. NY, YF, SZ, XG, YL, LL, LX, WW, CZ, and QL co‐organized the fasting practice, collected and prepared samples, and discussed the manuscript. JW conceived the study, and finalized the manuscript.

## Supporting information

Supplementary Material

## Data Availability

The data that support the findings of this study are available from the corresponding authors upon request.
