# Supplementary material for: Innate immune remodeling by short‐term intensive fasting
Source: Aging Cell. 2021 Oct 27;20(11):e13507. doi: 10.1111/acel.13507 (PMC8590100; doi:10.1111/acel.13507)
Supplement: Supplementary file 1 — Supplementary Material [file ACEL-20-e13507-s001.pdf]

Innate immune remodeling by short-term intensive fasting

Jiawei Qian, Yixuan Fang, Na Yuan, Xueqin Gao, Yaqi Lv, Chen Zhao, Suping Zhang, Quan Li, Lei Li, Li Xu, Wen Wei, Jianrong Wang

Supplementary information

Table S1. Information on the samples of the fasting subjects for multi-omics study

14 samples for multi-omics study from 11 subjects aged from 29-60 years old

Sample (0 hr)	Sample (72 hrs)	Subject label No	Gender	Age	Height(cm)	Weight(kg)
3-1	3-2	3	Male	43	171	60
49-1	49-2	49	Female	46	158	55.4
66-1	66-2	66	Female	40	N/A	77
88-1	88-2	88	Male	52	178	N/A
48-1	48-2	48	Male	44	179	85
86-1	86-2	86	Male	49	170	75.7
41-1	41-2	41	Female	29	162	N/A
12-3	12-4	12	Male	38	N/A	84
36-3	36-4	36	Female	N/A	N/A	N/A
50-3	50-4	50	N/A	47	N/A	N/A
52-3	52-4	52	Male	60	N/A	72

Red：samples with good quality and sufficient quantity, used for transcriptomic and proteomic sequencing
Red in green background：paired samples
Grey in white background: samples not used due to insufficiency in quantity and quality

Table S2. Information on the demographic data of the fasting subjects for lab biochemical analysis

57 subjects aged from 16-59 years old, provided samples for biochemical analysis; data is shown in Figure 4c,d in the main text

Subject	Gender	Age	Height (cm)	Weight (kg)
18	Female	**16**	N/A	73
83	Female	20	163	66.5
6	Male	21	178	N/A
27	Male	22	178	88
69	Female	27	163	N/A
81	Male	27	180	N/A
15	Female	28	N/A	N/A
34	Female	28	N/A	N/A
43	Female	28	166	N/A
37	Female	29	166	N/A
41	Female	29	162	N/A
79	Female	29	N/A	N/A
78	Male	31	N/A	N/A
24	Female	33	160	75
63	Female	34	163	N/A
9	Male	35	170	N/A
22	Female	36	160	48.1
64	Female	36	155	N/A
62	Male	37	180	N/A
5	Female	38	165	77
39	Female	38	160	N/A
23	Female	39	166	69
58	Female	39	N/A	N/A
96	Male	39	185	96
28	Female	40	170	N/A
66	Female	40	N/A	N/A
97	Female	40	170	65
4	Female	41	168	63
16	Male	42	180	95
99	Female	42	N/A	N/A
3	Male	43	171	60
36	Female	44	160	N/A
38	Male	44	170	N/A
48	Male	44	179	85
65	Female	44	N/A	N/A
47	Male	46	175	N/A
49	Female	46	158	55.4
87	Female	47	160	64
74	Female	48	160	58.1
85	Female	48	N/A	N/A
90	Male	48	N/A	N/A
46	Male	49	176	N/A
68	Male	49	171	N/A
86	Male	49	170	75.7
30	Female	50	N/A	60.5
40	Male	50	N/A	N/A
82	Female	50	165	57
42	Female	51	165	N/A
77	Male	51	165	77
72	Male	52	165	69.7
88	Male	52	N/A	65
101	Male	54	N/A	N/A
70	Male	55	N/A	N/A
32	Male	56	N/A	71
71	Female	58	166	69.5
73	Female	59	163	71.5
45	Male	**N/A**	173	N/A

Table S3. Information on the demographic data of the fasting subjects for blood routine test

40 subjects aged 27-67 years old, provided samples for biochemical analysis; data is shown in Supplementary Figure 3 in the Supplementary Information				
Subject	Gender	Age	Height (cm)	Weight (kg)
1	Male	56	171	86
9	Female	43	160	73
10	Female	44	160	73
11	Female	41	165	56
12	Male	61	170	72
13	Male	49	170	68
14	Male	31	177	72
15	Male	37	180	75
16	Male	31	183	100
17	Female	30	167	60
18	Female	49	160	57
19	Female	51	158	50
21	Female	64	158	63.3
22	Female	34	160	60
23	Female	59	158	70
24	Male	37	172	66
25	Female	37	160	62
26	Male	55	172	90
27	Male	50	163	75
28	Male	58	178	85
29	Female	57	162	65
30	Female	40	158	52.5
31	Female	32	162	53
32	Female	36	165	66
33	Male	46	163	60
34	Female	35	177	80
35	Female	47	167	51
36	Male	48	170	70
37	Male	33	180	82.5
38	Male	42	173	90
40	Female	27	169	81
41	Female	52	160	75
42	Female	41	162	60
43	Male	41	175	73
44	Female	40	155	60
46	Female	34	162	54
47	Male	32	180	90
70	Female	31	164	60
52	Female	67	158	70
53	Female	41	165	54

Table S4. Alignment results

Sample	Pair	Reads	Aligned 0 times	Aligned 0 times rate	Aligned exactly 1 time	Aligned exactly 1 time rate	Aligned >1 times	Aligned >1 times rate	Overall alignment rate
A1	no	22662170	516325	2.28%	19791348	87.33%	2354497	10.39%	97.72%
A2	no	22944961	640090	2.79%	19802001	86.30%	2502870	10.91%	97.21%
A3	control_1	22613370	522025	2.31%	19755640	87.36%	2335705	10.33%	97.69%
A4	control_2	22344990	566912	2.54%	19259982	86.19%	2518096	11.27%	97.46%
A5	control_3	22694000	516252	2.27%	19901274	87.69%	2276474	10.03%	97.73%
A6	control_4	22377726	550864	2.46%	19050697	85.13%	2776165	12.41%	97.54%
A7	no	22687315	475550	2.10%	19705008	86.85%	2506757	11.05%	97.90%
B1	no	22432770	579797	2.58%	19427278	86.60%	2425695	10.81%	97.42%
B2	treat_1	22479443	550117	2.45%	19517836	86.83%	2411490	10.73%	97.55%
B3	treat_2	22590793	571077	2.53%	19618550	86.84%	2401166	10.63%	97.47%
B4	treat_3	22593003	492670	2.18%	19758733	87.46%	2341600	10.36%	97.82%
B5	treat_4	22419674	479200	2.14%	19536577	87.14%	2403897	10.72%	97.86%
B6	no	22574387	547476	2.43%	19839813	87.89%	2187098	9.69%	97.57%
B7	no	22564598	532351	2.36%	19496114	86.40%	2536133	11.24%	97.64%

Table S5. Gene list for Figure S1c

Gene name for transcriptome						Gene name for proteome	
IGHV1-69	CYFIP1	TREM2	PIP4P2	RACK1	IGHV1-58	AIF1	DNM2
IGHV3-64	LRP1	IGHD	IGLV3-27	IL2RG	CDC42SE2	PAK1	AHSG
SYT7	IGLV3-25	IFNG	C2	NCK1	LDLR	FCGR3A	SNX3
IGHV1-2	IGHV3-48	IGHV3-33	DNM2	TLR2	SRC	TYROBP	MYO18A
IGHV3-13	IGKV1-16	CLN3	ACTG1	BECN1	AHSG	IGKV4-1	CYFIP1
XKR9	NR1H3	LIMK1	CD47	ACTR2	SIRPA	CD300A	SH3BP1
IGHV4-61	ABCA7	ADGRB1	IL15	ARPC1A	FPR2	ITGAM	LMAN2
CEBPE	UNC13D	IGHV1-3	MYO10	TICAM2	PPARG	CD14	APOA1
IGKV2D-28	DYSF	DOCK2	PTEN	ACTR3	IGHV5-10-1	ITGB2	PIK3R1
IGHV3-15	ANO6	ACTB	CDC42SE1	HSP90AA1	IL1B	ELMO2	ELMO1
ALOX15	IGLV3-1	PIK3R2	LYN	CD3G	RARA	ITGAL	NCKAP1L
IGLL5	ITGAL	TMEM175	PRKCE	RAB20	IGKV2D-40	NCF2	PLCG2
FCGR3A	FER1L5	CD300LF	IGLV2-8	IGHV4-31	MET	FYN	RAB34
IGHV3-43	XKR8	NCKIPSD	KIAA1109	ABI1	PRKCG	JMJD6	CYFIP2
MARCO	IGKV3-11	IGLV1-44	CLCN3	IGHV3-64D	F2RL1	FGR	C3
SYK	IGLV2-23	XKR6	IGHV3-66	JMJD6	TGM2	IGHA1	ARPC1A
CEACAM4	CLEC7A	PECAM1	ARPC4	FYN	RHOBTB1	NCKIPSD	CALR
IGHV2-70	MST1R	PTPRJ	NCKAP1	ANXA1	CCL2	CD247	RAP1A
IGKV4-1	IGHV3-74	IGHG1	IGHV1-69D	WIPF1	IGKV1D-33	RAB27A	HSP90AB1
PLD4	SPG11	ITGA2	IGHV3-38	CSK		VAMP7	MESD
GSN	SLC11A1	IGHV5-51	NCKAP1L	SPHK1		CDC42SE1	WAS
IGHV3-23	RHOBTB2	IGKV1-5	IGKV5-2	CORO1C		LYN	ACTB
IGLV1-51	IGHV1-18	IGKV2-30	MYH9	CDC42		BIN2	CORO1C
COLEC12	IGHV6-1	IGHV3-11	APPL1	CD93		C4A	BRK1
IGKV1D-39	ABL1	MFGE8	HMGB1	IL15RA		ARPC1B	CD3G
ITGAM	IGKV1-33	NCF4	PTX3	SOD1		TXNDC5	RAC1
CD36	IGKV1-12	IL2RB	RAB14	IGKV1D-12		TMEM175	ATG5
IGKV1-17	ELANE	WASF2	ELMO3	HSP90AB1		ITGB1	CORO1A
DOCK1	IGLV6-57	IGLL1	RAPGEF1	FCGR1A		PYCARD	HMGB1
LEPR	SIRPB1	ITGB1	PIKFYVE	WIPF2		ARPC3	MYH9
ITGB2	GATA2	IGHV4-28	PAK1	ATG5		ARHGAP25	RACK1
HAVCR1	IGKV1-39	RAC2	LMAN2	MERTK		PTPRJ	DOCK2
IGHV7-4-1	ARHGAP12	VAV1	IGKV3-15	ARPC1B		NCK1	ANXA11
IGHV3-21	IGLC3	HCK	IGKV2-28	CALR		WASF2	GRB2
IGKV3D-20	IGHV3-7	RAB11FIP2	PRKCD	ATG3		ACTR2	RAB7A
IGHV2-26	IGHV3-72	SYT11	FCN3	PLSCR1		HCK	RAB5A
TYROBP	C4A	CYFIP2	FCGR2A	PIK3R1		ACTR3	MYD88
FCER1G	XKR7	NCF2	ARPC5	RAB5A		IGHG2	RAB14
IGHA2	PLA2G6	IGKV1D-16	APPL2	CRK		TM9SF4	NCF4
IGLV2-14	IGLC2	ADORA1	EIF2AK1	STAP1		CYBA	PIK3R2
CD302	IGHV1-24	VAV2	GRB2	TULP1		ANXA1	ANO6
ANXA3	TUB	WASL	LYAR	TUSC2		ATG3	MAPK3
TXNDC5	RAB39A	PLCG1	SNX3	RAB34		RAC2	LYAR
BIN2	MYO7A	ARHGAP25	RAB27A	MESD		CDC42	ABI1
IGLV1-40	MYO18A	ELMO1	IGLV7-43	ELMO2		MAPK1	APPL1
IGHG4	IGHV2-5	IGLV2-11	RAB31	IRF8		ARPC5	PLCG1
SCARB1	IGHV4-39	ITGAV	CD247	IGHE		PIK3CA	APOA2
IGHV3-73	ICAM5	P2RY6	LEP	SLAMF1		RHOG	IL2RG
SFTPD	SIRPG	BAIAP2	RUBCN	IGHV4-34		MYO1G	SYK
FCN1	PRTN3	C3	ARPC2	IGHV1-45		RAPGEF1	RHOH
TYRO3	VAV3	IGHV3-53	PIK3CB	TM9SF4		WIPF1	MYO1C
GAS6	PLCG2	IGLV1-47	VAMP7	IGHV3-35		ICAM3	BECN1
IGHG2	IGHM	IGLV3-19	IGHV2-70D	C1orf43		UNC13D	GSN
IGHV4-59	CYBA	WIPF3	RHOH	ADIPOQ		PRKCD	ELANE
FCGR2B	IGKV3-20	IGLV3-21	APOA1	AZU1		CSK	AZU1
IGHA1	MAPK3	IGHV3-20	TLR4	FCN2		PECAM1	PRTN3
IGHV3-49	IGHV3-30	CD14	ADORA2A	RAB7A		IGHM	SPG11
CD300A	IGHV3-30	ITGB3	PTPRC	YES1		ARPC2	PTK2
C4B	IGHV1-46	PEAR1	ARPC3	THBS1		ARPC4	FCN1
APOA2	WAS	MAPK1	BRK1	RHOG		VAV1	ANXA3
MSR1	PLD2	ANXA11	TNF	PIK3CA		VAV2	DYSF
PIP5K1C	XKR4	CAMK1D	IGHV4-4	RAP1A		TREX1	SOD1
SH3BP1	ICAM3	MYO1G	IGKV2D-30	PIP5K1A		PTPRC	CD36
FGR	PLA2G5	MYD88	AXL	MYO1C		HSP90AA1	THBS1
RAB7B	LYST	AIF1	NOD2	TAFA4		PIP5K1C	ITGB3
PYCARD	IGHV1-69-2	CORO1A	RAC1	PTK2		CAMK1D	CRK

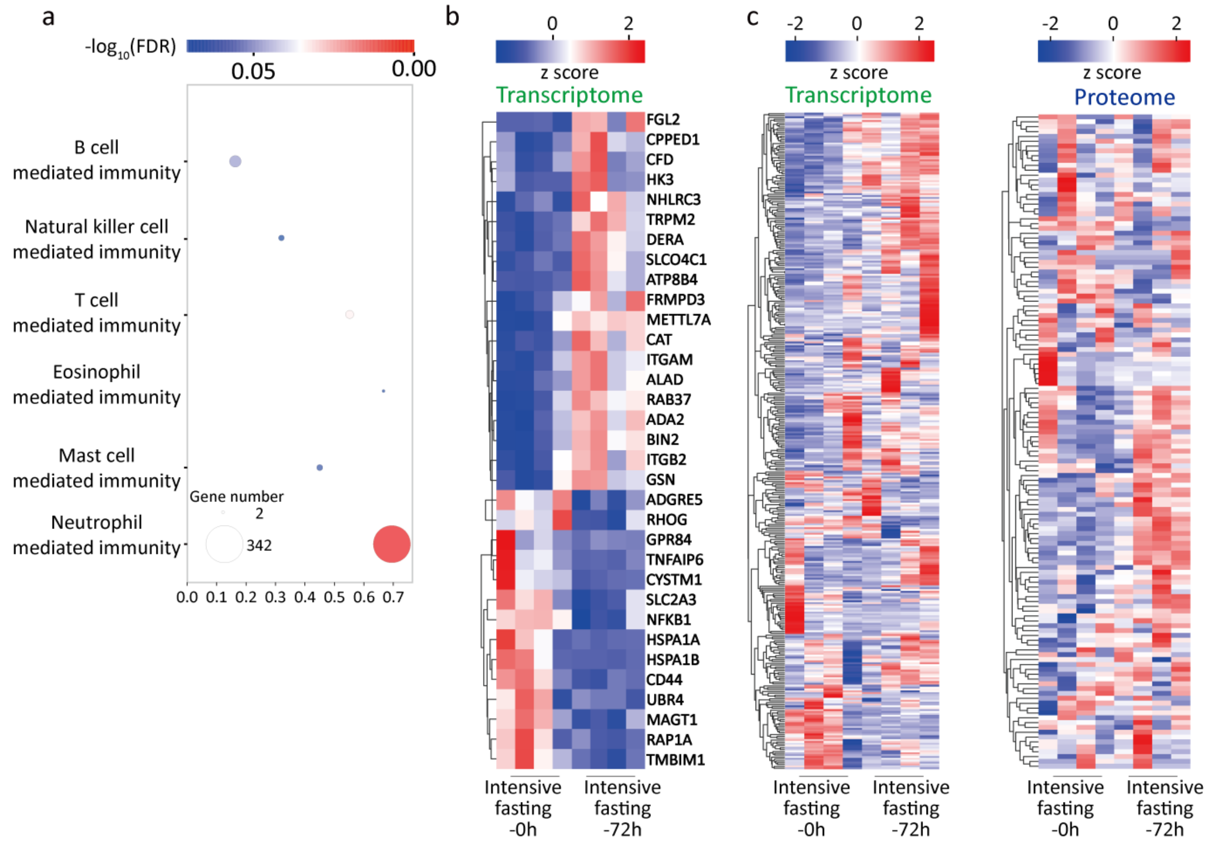

Figure S1. Neutrophil activation is the key immunological change in response to intensive fasting. a) Bubble plot depicting leukocyte functions of all proteins. Color bar represents the FDR of each enriched pathway, ranging from low to high, in correspondence to red to blue. **b)** Heatmap of genes related to neutrophil degranulation in transcriptome. Expression data was row normalized to illustrate the differential status. **c)** Heatmap of genes and protein expression related to phagocytosis. Expression data was row normalized to illustrate the differential status.

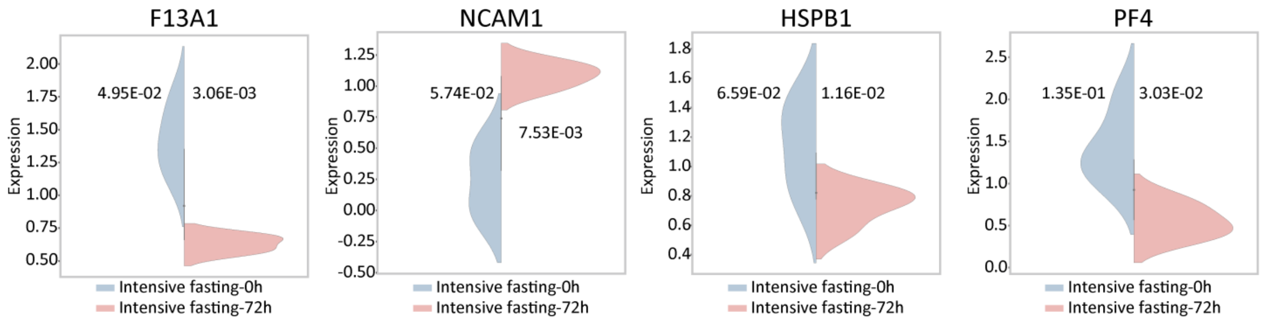

Figure S2. Intensive fasting may stabilize cytokine abundance Violin plot depicting cytokines that showed stabilized protein abundance. Gene expression were normalized. Variance of each gene in each study group is illustrated in the graph.

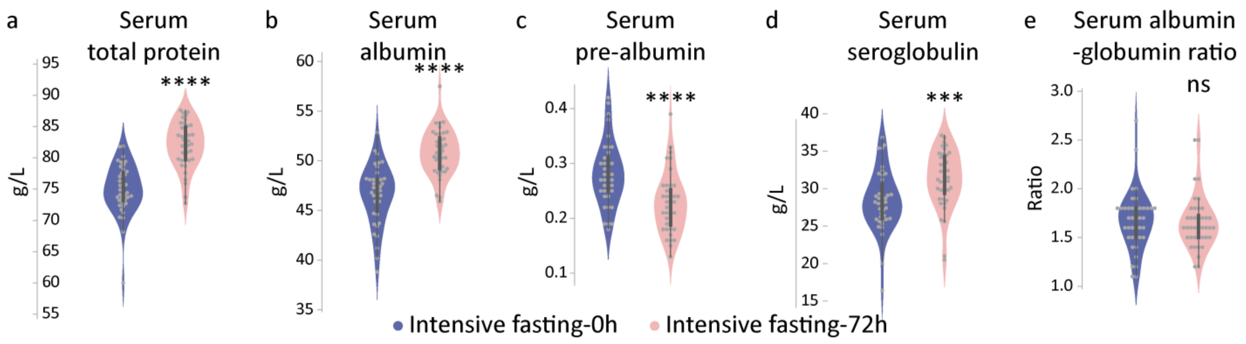

Figure S3. Serum level of total protein, albumin, pre-albumin, seroglobulin, albumin-globumin ratio before and after 72 hour intensive fasting. n = 40, paired Student's t test. This result was analyzed from the subjects participated in an earlier fasting event (Fang et al., BMJ Nutr Prev Health 2021;4(1):4-17)
